# Association between insulin resistance-related lipid indices and arthritis: a U. S. cross-sectional study

**DOI:** 10.3389/fnut.2025.1583598

**Published:** 2025-07-02

**Authors:** Ao Wu, Haodong Teng, Xiaoxia Wang, Ketao Shi, Zhi Gao, Honghao Xu, Liang Yue

**Affiliations:** ^1^The First Clinical College of Shandong University of Traditional Chinese Medicine, Jinan, China; ^2^Zhongshan Medical College of Sun Yat-sen University, Guangzhou, China; ^3^Affiliated Hospital of Shandong University of Traditional Chinese Medicine, Jinan, China; ^4^Anhui University of Traditional Chinese Medicine, Hefei, China

**Keywords:** TyG, insulin resistance, arthritis, cross-sectional study, NHANES

## Abstract

**Objectives:**

Arthritis is a degenerative disease that causes a huge social burden. Lipid-related molecules participate in the inflammatory response process of arthritis and are closely related to the pathological process of arthritis. Lipid-related indicators are easily available and have great potential in predicting arthritis. This study used cross-sectional data to explore lipid-related indicators and arthritis risk.

**Methods:**

18,683 participants were involved in this study, selected from the NHANES database covering the period from 2001 to 2018. The study utilized multivariate regression models to examine the association between various lipid-related parameters (including the TyG index, TyG-WC index, TyG-WHtR index, TyG-BMI index, HOMA-IR index, VAI index, and LAP index) and arthritis.

**Results:**

After taking into account and appropriately addressing potential confounding variables and factors, all seven lipid-related indicators were positively associated with arthritis risk, and there was a significant difference in the highest quartile of seven lipid-related indicators compared with the lowest quartile (*P* < 0.001). Among them, the area under the ROC curve (AUC) of TyG-WC, TyG-WHtR, TyG-BMI, and LAP was >0.6, indicating they had modest accuracy in predicting arthritis. Logistic regression analysis showed that the best Cut-off values for predicting arthritis for these indicators were as follows: TyG: 8.45 [Odds ratio (95% Cl) = 1.77 (1.62, 1.94)]; TyG-WC: 850.39 [Odds ratio (95% Cl) = 1.36 (1.24, 1.49)]; TyG-WHtR: 4.97 [Odds ratio (95% Cl) = 2.39 (2.17, 2.63)]; TyG-BMI: 255.24 [Odds ratio (95% Cl) = 1.87 (1.71, 2.05)]; HOMA-IR: 2.79 [Odds ratio (95% Cl) = 1.51 (1.39, 1.65)]; VAI: 1.35 [Odds ratio (95% Cl) = 1.60 (1.47, 1.75)]; LAP: 33.46 [Odds ratio (95% Cl) = 1.20 (1.09, 1.31)], both *P*-values are < 0.001.

**Conclusions:**

The results showed that seven lipid-related markers were positively associated with arthritis risk. Enhancing the management of glucose, lipids, and insulin sensitivity may significantly reduce the risk of arthritis.

## Introduction

Arthritis is an inflammatory degenerative disease, mainly composed of osteoarthritis (OA) and rheumatoid arthritis (RA). Arthritis is characterized by the involvement of one or more joints, which can lead to joint discomfort and dysfunction, thus significantly reducing the patient's quality of life (1). Arthritis is closely related to aging, and due to an aging population, the burden of arthritis on society is also increasing (2, 3). As stated by the World Health Organization (WHO), arthritis has emerged as a prevalent factor leading to disability in the United States. An Australian study shows that there were already 3.90 million arthritis patients in Australia in 2018, which is expected to increase to 5.40 million by 2030, and the cost of arthritis to the health system will also increase to $7.60 billion (4).

Given the significant harm it causes, it is crucial to conduct early screening for high-risk populations by identifying risk factors and implementing effective management strategies, which are important for both individuals and society.

Insulin resistance (IR) is an important cause of metabolic diseases (5). In recent years, insulin resistance and arthritis have been widely studied. Research by Hamada et al. (6) shows that insulin plays a protective and anti-inflammatory role in the joints, and IR will destroy this protective effect and cause joint damage. Some scholars have also shown that insulin resistance can reduce bone mineral density (7). The current studies on the association between insulin resistance and arthritis are mostly animal experiments, and there is a lack of large-scale population studies.

To investigate the association between insulin resistance and arthritis, this study used seven readily available lipid-related indices closely related to insulin resistance. The triglyceride-glucose (TyG) index is an emerging marker with widely recognized effects on insulin resistance (8). Body mass index (BMI), waist circumference (WC), and waist-to-height ratio (WHtR) were anthropometric measures used to assess obesity and metabolic risk. TyG-WC, TyG-WHtR, and TyG-BMI are derived indicators of TyG and are closely related to insulin resistance (9, 10). They are more accurate than TyG alone (9, 11). The Homeostatic model assessment of insulin resistance (HOMA-IR) is a commonly utilized indicator for assessing the degree of insulin resistance in individuals. Its widespread use is attributed to its readily accessible nature. HOME-IR ≥ 2.2 is diagnosed as IR. Visceral adiposity index (VAI) and lipid accumulation products (LAP) are also considered predictors of IR (12, 13). At present, there are few studies on lipid-related indicators and arthritis. The purpose of this study is to explore the diagnostic accuracy of lipid-related indicators in arthritis.

## 2 Materials and methods

### 2.1 Research participants

The National Health and Nutrition Examination Survey (NHANES) is a countrywide survey conducted by the National Center for Health Statistics (NCHS) to provide national health information. The NHANES data used in this study is available for public access at the following link: https://www.cdc.gov/nchs/nhanes/ (retrieved on June 14, 2024). A morally sound protocol that received approval from the NCHS Research Ethics Review Board was put into place, and all enrolled individuals consented by signing an informed consent form.

**Figure 1:**
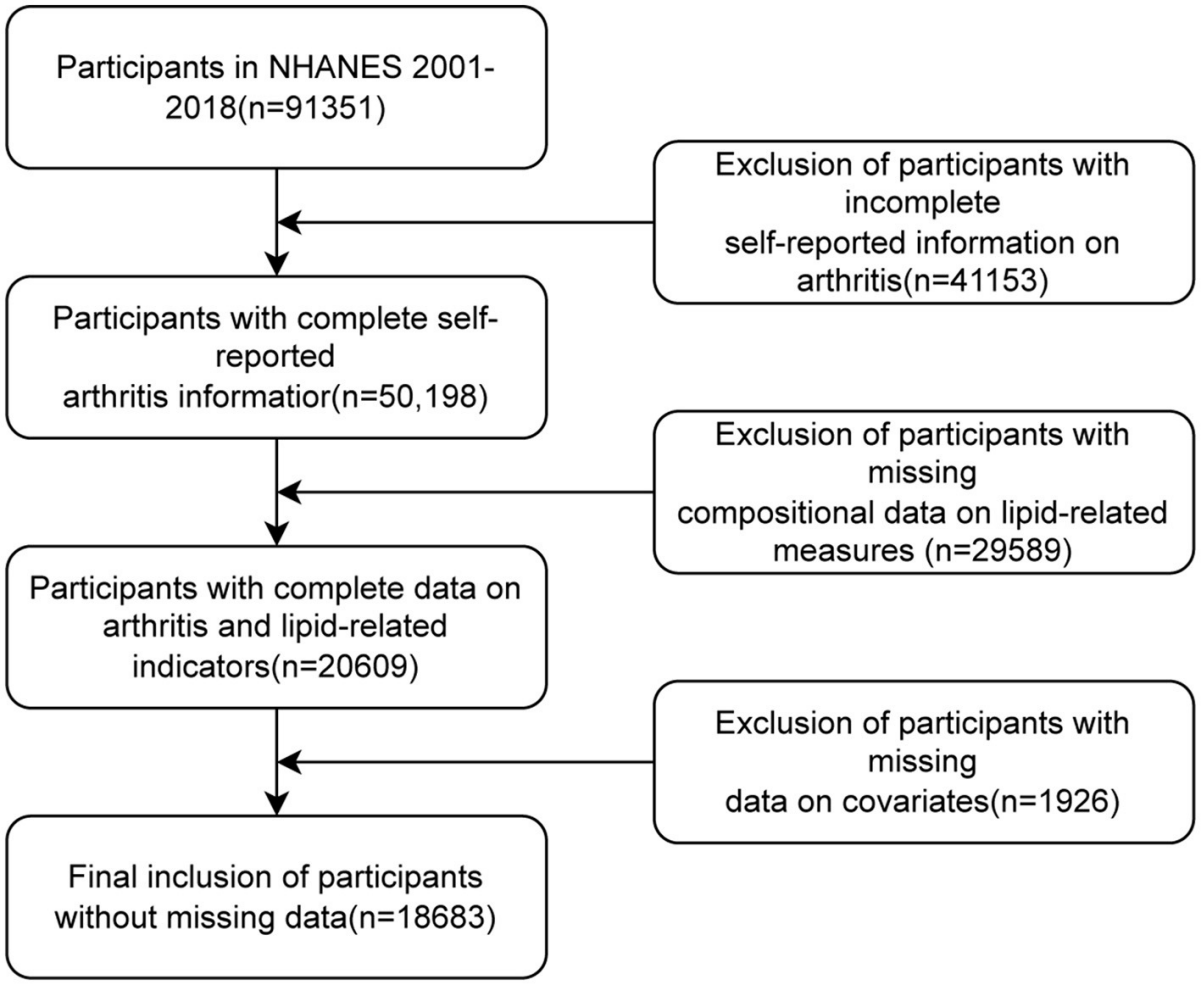
Participant screening flowchart.

In this study, we examined data from nine cycles of NHANES 2001–2018. A total of 91,351 participants were enrolled in these nine cycles of NHANES surveys. We first excluded patients with missing arthritis data (*n* = 41,153), further excluded participants with missing compositional data on lipid-related measures (*n* = 29,589), and finally excluded participants with missing covariate data (*n* = 1,926). Ultimately, 18,683 individuals were recruited for the study. Please refer to Figure 1 for the visual representation of the study's progression.

### 2.2 Measurement of lipid-related indicators

This study measured seven indicators (TyG, TyG-WC, TyG-WHtR, TyG-BMI, HOME-IR, VAI, and LAP). TyG is a proxy indicator for insulin resistance (IR), reflecting lipid-glucose dysregulation; TyG-WC combines IR with abdominal obesity (visceral fat), and waist circumference (WC) can independently predict metabolic risk; TyG-WHtR adjusts waist circumference based on height, making it more sensitive than BMI for assessing cardiovascular metabolic risk; TyG-BMI combines IR with general obesity, making it more accurate and effective in assessing insulin resistance; HOME-IR is the gold standard for assessing IR; VAI quantifies visceral fat dysfunction and is used to predict metabolic syndrome; LAP reflects lipid accumulation in adipose tissue and serves as an early marker of IR in non-obese individuals. Examination data were collected: waist circumference (WC), Height, and BMI, and laboratory data were collected: Waist Circumference (WC): Measured at the iliac crest (NHANES protocol). Height: Measured via stadiometer. Subjects fasted for 9–12 h before blood collection, and total cholesterol (TC), fasting plasma glucose (FPG), fasting insulin (FINS), high-density lipoprotein cholesterol (HDL-c), low-density lipoprotein cholesterol (LDL-c) and triglycerides (TG) were collected. The calculation formula is as follows:

TyG = Ln [TG (mg/dl) × FPG (mg/dl)/2]
TyG-WC = TyG × waist circumference (cm)
TyG-WHtR = TyG × waist circumference (cm)/Height (cm)
TyG-BMI = TyG × BMI
HOME-IR = FINS (μU/ml) × FPG (mmol/L)/22.5
VAI for men = [waist circumference (cm)/(39.69 + 1.88 × BMI)] × triglycerides (mmol/L)/1.03 × [1.31/HDL-c (mmol/L)]
VAI for women = [waist circumference (cm)/(36.58 + 1.89 × BMI)] × triglycerides (mmol/L)/0.81 × [1.51/HDL-c (mmol/L)]
LAP for men = [waist circumference (cm) – 65] × [triglycerides (mmol/L)]
LAP for women = [waist circumference (cm) – 58] × [triglycerides (mmol/L)]

### 2.3 Assessment of arthritis

Self-reported arthritis diagnoses were determined by the following questions: “Has a doctor or other health professional ever told you that you had arthritis?”, if the participant answered “Yes”, they were diagnosed with arthritis. Previous scholars have shown that self-reported arthritis has an 85% concordance rate with clinically diagnosed arthritis (14), suggesting that self-reported arthritis results are highly credible.

### 2.4 Covariates

Several potential confounding factors were included, based on research by previous scholars. Demographic information was collected, including age, sex, and ethnicity. Age was used as a continuous variable. Race was categorized into five groups: Mexican American, Other Hispanic, Non-Hispanic White, Non-Hispanic Black, and Other Race categories were included in the classification system. Educational achievement was categorized as: “Less Than 9th Grade”, “9–11th Grade”, “High School Grad/GED”, “Some College or AA degree”, and “College Graduate or above”. BMI was classified as underweight (BMI < 18.5), normal weight (18.5–25), overweight (25–30), and obesity (BMI > 30), according to the World Health Organization's definition of BMI thresholds. According to the answer to the question “Have you smoked at least 100 cigarettes in your entire life?” smoking was classified as “Yes” and “No”. Hypertension was defined as the answer to the question: “Have you ever been told by a doctor or other health professional that you had hypertension, also called high blood pressure?”, divided into two groups (Yes or No). According to “Other than during pregnancy, have you ever been told by a doctor or health professional that you have diabetes or sugar diabetes?” The answer to this question divides diabetes into two groups (Yes or No). Coronary heart disease and stroke can be characterized through the following inquiries: “Has a doctor or other health professional ever told you that you had coronary heart disease?” “Has a doctor or other health professional ever told you that you had a stroke?” both divided into two groups (Yes or No).

### 2.5 Statistical analysis

We conducted the statistical data analysis with the R software (version 4.4.0), and to ensure that the analysis results were nationally representative, data analytics took into account sampling weights. Participants were divided into two groups (Yes or No) based on whether or not they had arthritis, and an analysis of baseline characteristics was performed. Continuous variables were expressed using the Wilcoxon rank sum test, using the median (interquartile range, IQR). Classifying variables were described using the chi-square test, and proportions were used to describe classifying variables. Participants were divided into two groups (Yes or No) for baseline analysis to compare the differences between various variables in arthritis and IR. Subsequently, the interquartile of seven lipid-related indicators were calculated, and the independent association between lipid-related indicators and arthritis was assessed using multivariate logistic regression analysis and covariate-corrected models. Then, using lipid-related measures as continuous variables, a restricted cube plot was drawn to assess the non-linear relationship between lipid-related measures and arthritis risk, adjusting for confounding factors. An ROC curve was constructed and the AUC was utilized to evaluate and compare the diagnostic precision of various lipid-related parameters in detecting insulin resistance and arthritis, to demonstrate that lipid-related measures are closely related to IR, and to evaluate high-efficiency measures for predicting arthritis. When the Youden index (sensitivity + specificity – 1) was the largest, the value of the index was the critical value. Logistic regression was conducted to assess the odds ratio (OR) of arthritis and insulin resistance (IR) occurring when the lipid-related index surpassed the critical threshold. Statistical significance was determined at a *P*-value below 0.05. Analyses utilized R's survey package to account for NHANES sampling weights, stratification, and clustering. ROC curves were generated using weighted sensitivity/specificity to ensure nationally representative performance estimates. Covariates were selected a priori based on their established roles as confounders in arthritis and metabolic disease literature (1, 3, 6). Model 1 examined unadjusted associations. Model 2 adjusted for core demographic variables (sex, age, race) to account for non-modifiable risks. Model 3 further adjusted for socioeconomic factors (education), cardiometabolic comorbidities (coronary heart disease, stroke, hypertension), lifestyle (smoking), and biomarkers (TC, AST, ALT) to isolate the independent association between lipid indices and arthritis. We avoided adjusting for BMI or diabetes to prevent overadjustment, as these are integral to lipid indices or share causal pathways with the exposure. Sensitivity analyses confirmed model robustness.

## 3 Results

### 3.1 Baseline characteristics

The baseline characteristics according to the prevalence of arthritis were shown in Table 1. 18,683 participants were included in this study, of which 52% were women and 48% were men. The overall prevalence of arthritis was 25%. As can be seen from the table, the risk of arthritis was positively correlated with age, and the prevalence was higher in women. The prevalence of non-Hispanic White participants was higher relative to other ethnicities. In arthritic participants, the median of all seven metrics was greater than in non-arthritic participants (*p* < 0.001). The table of baseline characteristics grouped according to prevalence or absence of insulin resistance was shown in Table 2, and the median of the seven metrics was similarly greater in insulin-resistant participants than in non-insulin-resistant participants.

**Table 1:** Baseline characteristics of participants in the NHANES 2001–2018 cycle according to whether they had arthritis.

**Characteristics**			**Arthritis**		***P* value**
	***N***	**Overall**, ***N*** = **18,683 (100%)**	**No**, ***N*** = **13,649 (75%)**	**Yes**, ***N*** = **5,034 (25%)**	
Age, years	18,683	46 (33, 59)	41 (30, 53)	59 (49, 69)	<0.001
**Sex**					
Female	18,683	9,732 (52%)	6,759 (49%)	2,973 (60%)	<0.001
Male		8,951 (48%)	6,890 (51%)	2,061 (40%)	
**Race**					
Non-Hispanic White	18,683	8,356 (68%)	5,588 (65%)	2,768 (77%)	<0.001
Non-Hispanic Black		3,652 (11%)	2,663 (12%)	989 (9.9%)	
Mexican American		3,181 (8.3%)	2,585 (9.6%)	596 (4.3%)	
Other Race		1,852 (7.1%)	1,548 (7.5%)	304 (3.1%)	
Other Hispanic		1,642 (5.3%)	1,265 (5.9%)	377 (3.3%)	
**Education**					
Less than 9th grade	18,683	2,065 (5.7%)	1,399 (5.3%)	666 (6.9%)	<0.001
9–11th grade		2,652 (11%)	1,879 (10%)	773 (13%)	
High school Grad/GED		4,295 (24%)	3,057 (23%)	1,238 (26%)	
Some college or AA degree		5,394 (31%)	3,961 (31%)	1,433 (31%)	
College graduate or above		4,277 (29%)	3,353 (30%)	924 (23%)	
Smoke	18,683	8,414 (46%)	5,689 (42%)	2,725 (55%)	<0.001
**BMI**					
Underweight	18,683	296 (1.7%)	243 (1.9%)	53 (1.1%)	<0.001
Normal weight		5,323 (30%)	4,255 (33%)	1,068 (22%)	
Overweight		6,336 (33%)	4,712 (34%)	1,624 (31%)	
Obesity		6,728 (35%)	4,439 (32%)	2,289 (45%)	
Diabetes	18,683	2,157 (8.5%)	1,143 (6.1%)	1,014 (15%)	<0.001
Hypertension	18,683	6,514 (31%)	3,634 (24%)	2,880 (52%)	<0.001
Coronary heart disease	18,683	748 (3.4%)	341 (2.1%)	407 (7.1%)	<0.001
Stroke	18,683	655 (2.7%)	280 (1.6%)	375 (6.1%)	<0.001
TC (mg/dl)	18,683	190 (165, 217)	189 (164, 215)	194 (168, 223)	<0.001
TG (mg/dl)	18,683	103 (71, 150)	99 (68, 145)	114 (81, 166)	<0.001
Glu (mg/dl)	18,683	99 (92, 107)	98 (91, 105)	103 (95, 114)	<0.001
INS (μU/ml)	18,683	9 (6, 15)	9 (6, 14)	10 (6, 17)	<0.001
AST (U/L)	18,683	22 (19, 27)	22 (19, 27)	23 (19, 27)	<0.001
ALT (U/L)	18,683	21 (16, 28)	21 (16, 29)	21 (16, 28)	0.2
HDL (mmol/L)	18,683	1.34 (1.11, 1.63)	1.32 (1.11, 1.63)	1.34 (1.11, 1.66)	0.012
LDL (mmol/L)	18,683	2.90 (2.33, 3.52)	2.90 (2.33, 3.52)	2.92 (2.33, 3.57)	0.3
TyG	18,683	8.55 (8.14, 8.98)	8.50 (8.09, 8.92)	8.71 (8.31, 9.12)	<0.001
TyG-WC	18,683	835 (719, 958)	814 (702, 934)	898 (783, 1,019)	<0.001
TyG-WHtR	18,683	4.95 (4.28, 5.67)	4.81 (4.15, 5.50)	5.35 (4.70, 6.08)	<0.001
TyG-BMI	18,683	238 (200, 283)	232 (196, 275)	257 (216, 306)	<0.001
HOMA-IR	18,683	2.23 (1.37, 3.87)	2.12 (1.32, 3.59)	2.65 (1.54, 4.78)	<0.001
VAI	18,683	1.41 (0.86, 2.37)	1.34 (0.82, 2.24)	1.64 (1.01, 2.75)	<0.001
LAP	18,683	41 (23, 72)	38 (20, 66)	53 (32, 87)	<0.001

Continuous variables were expressed as medians (interquartile range, IQR) and categorical variables as proportions (%) and analyzed using sampling weights.

**Table 2:** Baseline characteristics of NHANES 2001–2018 cycle participants by presence or absence of insulin resistance.

**Characteristics**			**Insulin resistance**		***P* value**
	***N***	**Overall**, ***N*** = **18,683 (100%)**	**IR**, ***N*** = **9,468 (48%)**	**Non-IR**, ***N*** = **9,215 (52%)**	
Age, years	18,683	46 (33, 59)	48 (35, 61)	44 (31, 57)	<0.001
**Sex**					
Female	18,683	9,732 (52%)	4,820 (49%)	4,912 (54%)	<0.001
Male		8,951 (48%)	4,648 (51%)	4,303 (46%)	
**Race**					
Non-Hispanic White	18,683	8,356 (68%)	3,871 (65%)	4,485 (71%)	<0.001
Non-Hispanic Black		3,652 (11%)	1,911 (12%)	1,741 (10%)	
Mexican American		3,181 (8.3%)	1,893 (10%)	1,288 (6.4%)	
Other Race		1,852 (7.1%)	865 (5.5%)	987 (5.5%)	
Other Hispanic		1,642 (5.3%)	928 (5.8%)	714 (4.7%)	
**Education**					
Less Than 9th Grade	18,683	2,065 (5.7%)	1,209 (6.8%)	856 (4.7%)	<0.001
9–11th Grade		2,652 (11%)	1,414 (12%)	1,238 (10%)	
High School Grad/GED		4,295 (24%)	2,259 (26%)	2,036 (22%)	
Some College or AA degree		5,394 (31%)	2,749 (32%)	2,645 (30%)	
College Graduate or above		4,277 (29%)	1,837 (24%)	2,440 (33%)	
Smoke	18,683	8,414 (46%)	4,232 (46%)	4,182 (46%)	0.7
**BMI**					
Underweight	18,683	296 (1.7%)	29 (0.3%)	267 (2.9%)	<0.001
Normal weight		5,323 (30%)	1,219 (12%)	4,104 (46%)	
Overweight		6,336 (33%)	3,289 (33%)	3,047 (33%)	
Obesity		6,728 (35%)	4,931 (54%)	1,797 (18%)	
Diabetes	18,683	2,157 (8.5%)	1,486 (12%)	671 (5.2%)	<0.001
Hypertension	18,683	6,514 (31%)	3,991 (39%)	2,523 (24%)	<0.001
Coronary heart disease	18,683	748 (3.4%)	471 (4.5%)	277 (2.3%)	<0.001
Stroke	18,683	655 (2.7%)	379 (3.2%)	276 (2.3%)	0.002
AR	18,683	5,034 (25%)	2,855 (29%)	2,179 (22%)	<0.001
TC (mg/dl)	18,683	190 (165, 217)	192 (166, 220)	189 (164, 215)	<0.001
TG (mg/dl)	18,683	103 (71, 150)	124 (87, 175)	87 (62, 123)	<0.001
Glu (mg/dl)	18,683	99 (92, 107)	104 (97, 114)	95 (89, 101)	<0.001
INS (μU/ml)	18,683	9 (6, 15)	14 (11, 19)	6 (4, 8)	<0.001
AST (U/L)	18,683	22 (19, 27)	22 (19, 27)	22 (19, 26)	<0.001
ALT (U/L)	18,683	21 (16, 28)	23 (17, 31)	19 (15, 25)	<0.001
HDL (mmol/L)	18,683	1.34 (1.11, 1.63)	1.22 (1.03, 1.45)	1.47 (1.22, 1.78)	<0.001
LDL (mmol/L)	18,683	2.90 (2.33, 3.52)	2.97 (2.40, 3.60)	2.85 (2.30, 3.47)	<0.001
TyG	18,683	8.55 (8.14, 8.98)	8.80 (8.42, 9.17)	8.32 (7.97, 8.71)	<0.001
TyG_WC	18,683	835 (719, 958)	926 (832, 1,026)	749 (662, 851)	<0.001
TyG_WHtR	18,683	4.95 (4.28, 5.67)	5.47 (4.93, 6.07)	4.43 (3.93, 5.02)	<0.001
TyG_BMI	18,683	238 (200, 283)	271 (237, 310)	210 (183, 243)	<0.001
HOMA_IR	18,683	2.23 (1.37, 3.87)	3.68 (2.80, 5.21)	1.40 (0.99, 1.80)	<0.001
VAI	18,683	1.41 (0.86, 2.37)	1.88 (1.19, 2.91)	1.08 (0.70, 1.74)	<0.001
LAP	18,683	41 (23, 72)	60 (39, 91)	27 (16, 47)	<0.001

Continuous variables were expressed as medians (interquartile range, IQR) and categorical variables as proportions (%) and analyzed using sampling weights.

### 3.2 Association of lipid-related markers with arthritis

**Figure 2:**
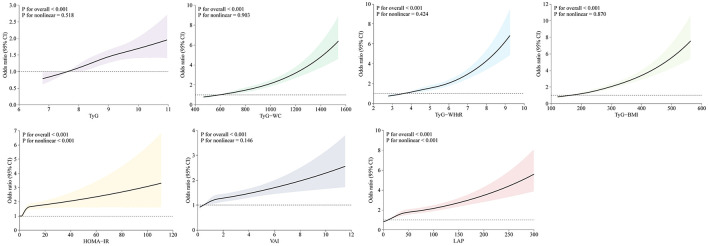
Restricted cubic splines reflected the dose-response relationship between lipid-related markers and arthritis. Models were adjusted for sex, age, race, education, coronary heart disease, stroke, smoke, hypertension, TC, AST, and ALT. Horizontal coordinates represent the seven lipid-related indices and vertical coordinates represent the ORs for arthritis. An overall *P* value of < 0.001 indicates a significant association and a non-linear association *P* value >0.05 indicates a linear dose-response relationship.

The association of lipid-related indices (continuous variables) with arthritis was shown in Figure 2, which adjusted for covariates (sex, age, race, education, coronary heart disease, stroke, smoke, hypertension, TC, AST, and ALT), and the results showed that TyG, TyG- WC, TyG-WHtR, TyG-BMI, and VAI were all linearly associated with arthritis risk (*P* for non-linear>0.05), while HOME-IR and LAP were non-linearly associated with arthritis risk (*P* for non-linear < 0.001). The non-linear *P*-value >0.05 confirms that for TyG, TyG-WC, TyG-WHtR, TyG-BMI, and VAI, arthritis risk increases in a proportional, linear manner as the index rises. This supports using these indices as straightforward, scalable predictors in clinical practice.

The relationship between lipid-related measures and arthritis was examined using multivariate logistic regression analysis. The results were shown in Table 3. After making adjustments for all covariates in the analysis and ensuring that potential confounding factors have been accounted for, Q4 of the seven lipid-related measures significantly differed from Q1 (*P* < 0.001), and arthritis risk showed a positive association with measures related to lipids. TyG-WC, TyG-WHtR, and TyG-BMI were found to be more effective predictors compared to the TyG index alone.

**Table 3:** Multivariate logistic regression analysis of lipid-related indexes and arthritis.

**Index**	**Crude OR (95% CI)**	***P*-value**	**Model 1 OR (95% CI)**	***P*-value**	**Model 2 OR (95% CI)**	***P*-value**
**TyG**						
Q1	—	—	—	—	—	—
Q2	1.51(1.31, 1.74)	<0.001	1.22(1.05, 1.42)	0.010	1.14(0.98, 1.34)	0.10
Q3	1.93(1.68, 2.23)	<0.001	1.45(1.25, 1.69)	<0.001	1.31(1.11, 1.54)	0.001
Q4	2.54(2.22, 2.90)	<0.001	1.79(1.54, 2.08)	<0.001	1.52(1.30, 1.78)	<0.001
**TyG-WC**						
Q1	—	—	—	—	—	—
Q2	1.82(1.58, 2.09)	<0.001	1.39(1.18, 1.64)	<0.001	1.33(1.13, 1.56)	<0.001
Q3	2.30(2.03, 2.61)	<0.001	1.72(1.50, 1.97)	<0.001	1.59(1.39, 1.82)	<0.001
Q4	3.53(3.06, 4.06)	<0.001	2.71(2.33, 3.16)	<0.001	2.28(1.95, 2.67)	<0.001
**TyG-WHtR**						
Q1	—	—	—	—	—	—
Q2	1.88(1.65, 2.16)	<0.001	1.29(1.09, 1.51)	0.003	1.22(1.04, 1.44)	0.017
Q3	2.51(2.17, 2.89)	<0.001	1.60(1.37, 1.85)	<0.001	1.45(1.25, 1.69)	<0.001
Q4	4.24(3.70, 4.84)	<0.001	2.54(2.19, 2.94)	<0.001	2.11(1.82, 2.46)	<0.001
**TyG-BMI**						
Q1	—	—	—	—	—	—
Q2	1.54(1.35, 1.77)	<0.001	1.25(1.07, 1.45)	0.005	1.21(1.04, 1.41)	0.016
Q3	1.75(1.52, 2.00)	<0.001	1.47(1.25, 1.72)	<0.001	1.38(1.18, 1.61)	<0.001
Q4	2.87(2.51, 3.29)	<0.001	2.70(2.33, 3.12)	<0.001	2.37(2.04, 2.74)	<0.001
**HOMA-IR**						
Q1	—	—	—	—	—	—
Q2	1.09 (0.95, 1.25)	0.2	1.00 (0.87, 1.16)	>0.9	0.97 (0.84, 1.13)	0.7
Q3	1.34 (1.19, 1.50)	<0.001	1.21 (1.06, 1.38)	0.004	1.11 (0.98, 1.27)	0.10
Q4	2.01 (1.75, 2.30)	<0.001	1.88 (1.62, 2.17)	<0.001	1.61 (1.39, 1.86)	<0.001
**VAI**						
Q1	—	—	—	—	—	—
Q2	1.34 (1.18, 1.53)	<0.001	1.23 (1.07, 1.41)	0.003	1.14 (1.0, 1.31)	0.060
Q3	1.64 (1.45, 1.86)	<0.001	1.38 (1.21, 1.57)	<0.001	1.22 (1.07, 1.41)	0.005
Q4	2.08 (1.85, 2.35)	<0.001	1.77 (1.55, 2.02)	<0.001	1.47 (1.28, 1.69)	<0.001
**LAP**						
Q1	—	—	—	—	—	—
Q2	1.87 (1.65, 2.11)	<0.001	1.40 (1.23, 1.60)	<0.001	1.35 (1.18, 1.54)	<0.001
Q3	2.34 (2.03, 2.71)	<0.001	1.63 (1.38, 1.91)	<0.001	1.50 (1.27, 1.76)	<0.001
Q4	3.33 (2.93, 3.80)	<0.001	2.38 (2.07, 2.74)	<0.001	2.05 (1.76, 2.38)	<0.001

OR, odds ratio; 95% CI, 95% confidence intervals.

Crude: unadjusted covariates.

Model 1: adjusted for gender, age and race.

Model 2: adjusted for gender, age, race, education, coronary heart disease, stroke, smoke, hypertension, TC, AST and ALT.

### 3.3 Predictive analysis of lipid-related indicators

**Figure 3:**
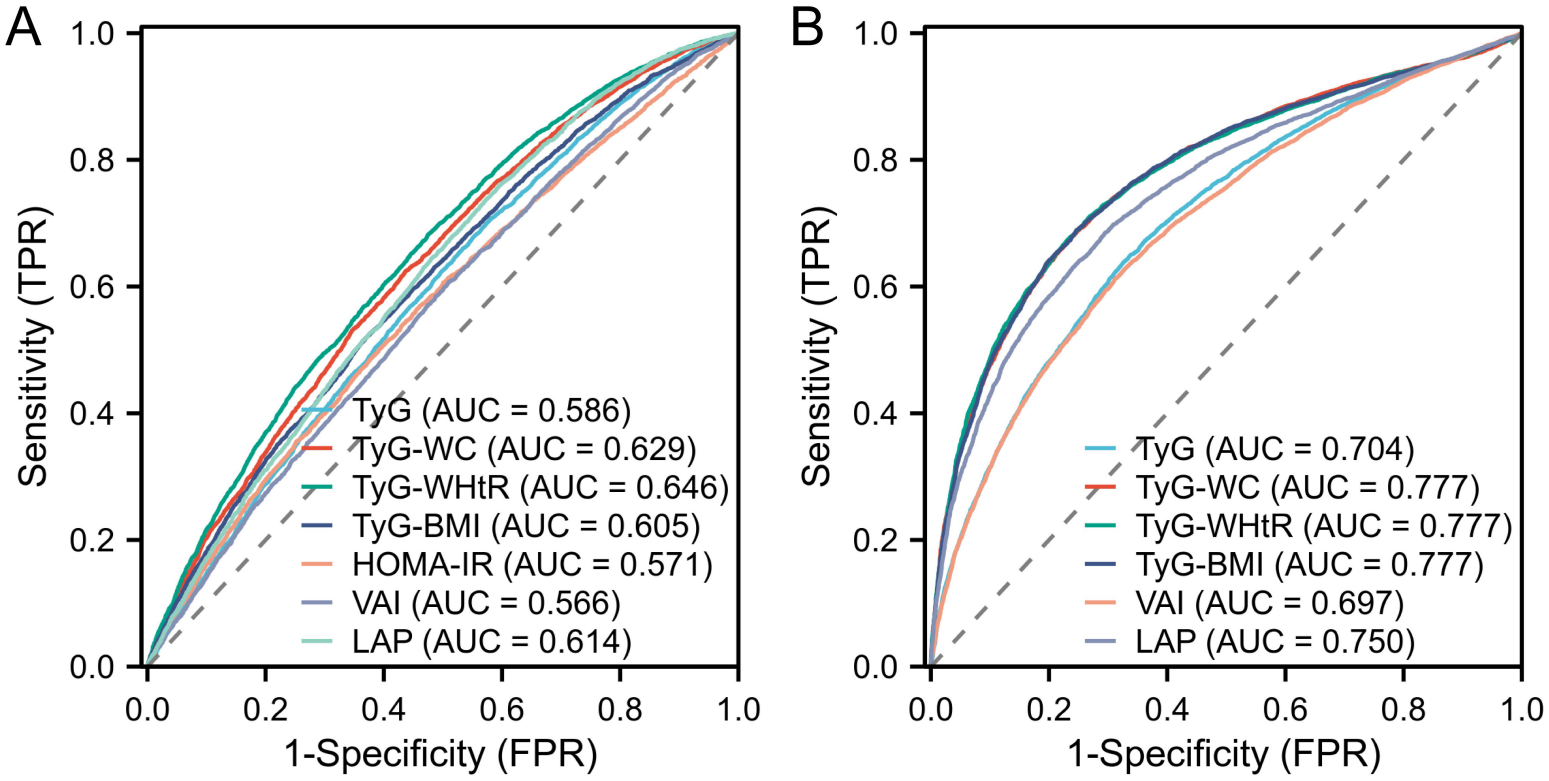
Displays the ROC curve utilized in assessing the predictive capability of lipid-related markers for arthritis and IR. In particular, **(A)** illustrates the ROC curve concerning 7 lipid-related indicators for arthritis evaluation, while (B) illustrates the ROC curve for the evaluation of IR based on 6 lipid-related indicators, with the exclusion of HOME-IR.

The performance of lipid-related indicators in predicting arthritis and IR was assessed using the ROC curve. Because the diagnosis of IR was defined according to HOME-IR ≥ 2.2, the prediction of IR excluded HOME-IR indicators. The findings were displayed in Figure 3. Figure 3A shows the ROC curve of lipid-related indicators predicting arthritis. Among them, the AUC of TyG-WC, TyG-WHtR, TyG-BMI, and LAP was >0.6, indicating that these four indicators have modest accuracy in predicting arthritis. Compared with the predictive power of lipid-related indicators for IR, the AUC of 6 indicators was >0.6, and even the AUC of TyG-WC, TyG-WHtR, TyG-BMI, and LAP was >0.75, indicating that the seven lipid-related indicators were closely related to IR. This further demonstrates the close relationship between arthritis and IR.

**Figure 4:**
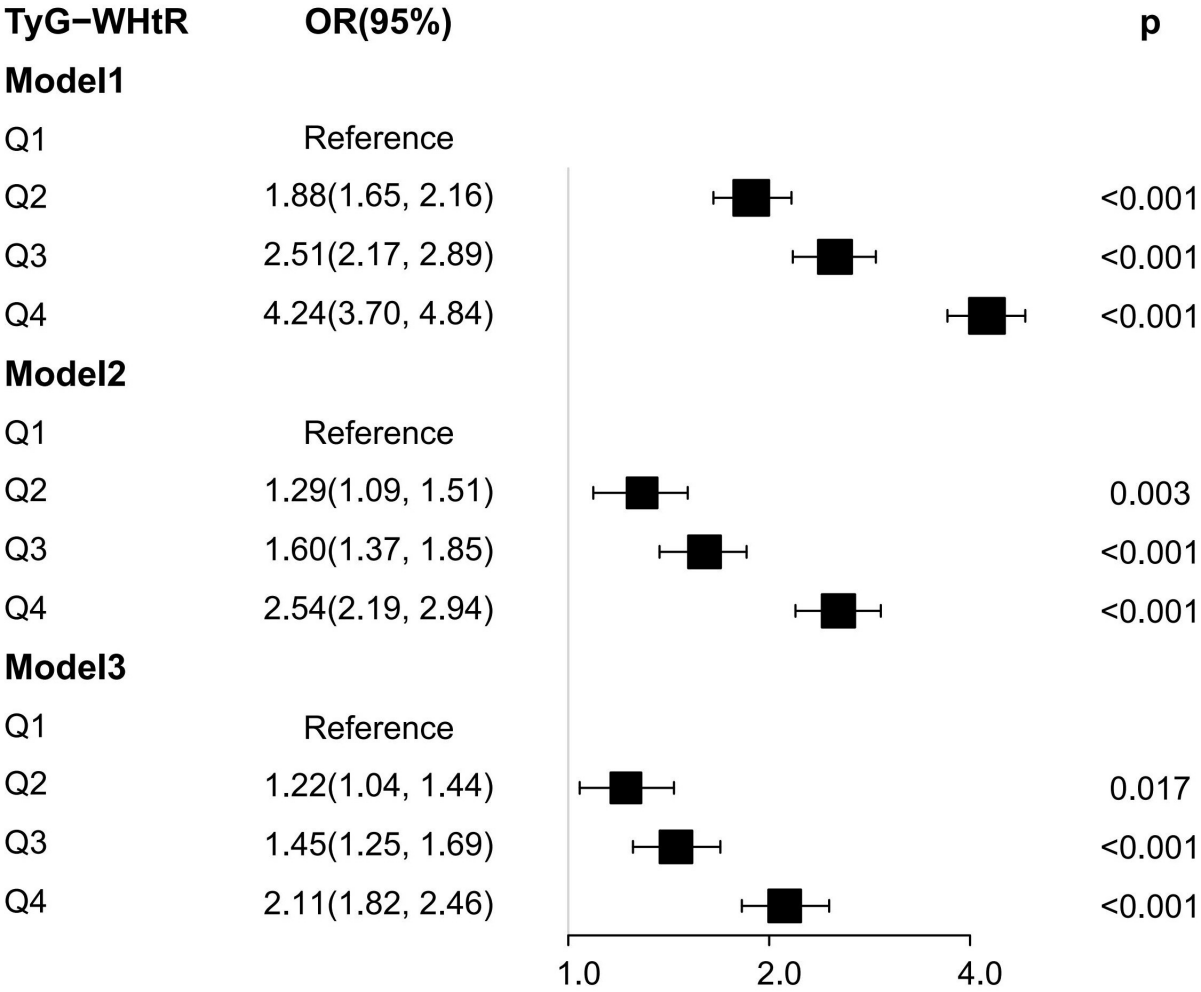
Logistic regression analysis plot of TyG-WHtR. OR: odds ratio, 95% CI: 95% confidence interval, Model 1:unadjusted, Model 2: adjusted for sex, age and race, Model 3: adjusted for sex, age, race, education, coronary heart disease, stroke, smoke, hypertension, TC, AST, and ALT.

We also calculated the OR values of the critical and critical values in arthritis and IR, and the results were shown in Table 4. The essential values of the TyG index, TyG-WC index, TyG-WHtR index, TyG-BMI index, HOMA-IR index, VAI index, and LAP index for predicting arthritis are 8.45, 850.39, 4.97, 255.24, 2.79, 1.35 and 33.46, respectively. The critical values of the TyG index, TyG-WC index, TyG-WHtR index, TyG-BMI index, VAI index, and LAP index for predicting IR are 8.55, 828.74, 4.92, 235.6, 1.34 and 41.66. It can be seen that the critical values of TyG, TyG-WHtR, and VAI indicators for arthritis and IR are almost the same, confirming that the two diseases are closely related. At the same time, Logistic regression analysis was performed on the threshold value of the index. The results showed that the OR value of TyG-WHtR was the most dominant in arthritis (OR = 2.39, 95% CI: 2.17, 2.63), TyG-WHtR has the best diagnostic performance for arthritis, and subsequently, we show the Logistic regression analysis plot of TyG-WHtR in Figure 4. While in IR, TyG-WHtR (OR = 7.68, 95% CI: 7.08, 8.50) and TyG-BMI (OR = 7.73, 95% CI: 7.03, 8.50) were the most dominant.

**Table 4:** In arthritis and IR, cut-off values for each parameter and their corresponding sensitivity, specificity, and odds ratios.

**Index**	**Arthritis**			**Insulin resistance**		
	**Cut-off values (sensitivity and specificity)**	**Odds ratio (95% Cl)**	***P*** **value**	**Cut-off values (sensitivity and specificity)**	**Odds ratio (95% Cl)**	***P*** **value**
TyG	8.45 (68.02, 44.64)	1.77 (1.62, 1.94)	<0.001	8.55 (63.79, 67.46)	3.80 (3.52, 4.10)	<0.001
TyG-WC	850.39 (62.38, 56.38)	1.36 (1.24, 1.49)	<0.001	828.74 (68.75, 75.25)	2.96 (2.71, 3.23)	<0.001
TyG-WHtR	4.97 (68.22, 52.52)	2.39 (2.17, 2.63)	<0.001	4.92 (67.36, 76.90)	7.68 (7.08, 8.50)	<0.001
TyG-BMI	255.24 (51.87, 63.10)	1.87 (1.71, 2.05)	<0.001	235.6 (68.88, 75.38)	7.73 (7.03, 8.50)	<0.001
HOMA-IR	2.79 (50.04, 60.85)	1.51 (1.39, 1.65)	<0.001	–	–	–
VAI	1.35 (60.69, 49.06)	1.60 (1.47, 1.75)	<0.001	1.34 (61.35, 68.71)	3.76 (3.50, 4.04)	<0.001
LAP	33.46 (73.70, 42.75)	1.20 (1.09, 1.31)	<0.001	41.66 (68.48, 70.55)	2.29 (2.12, 2.47)	<0.001

## 4 Discussion

In this research, we explored the association between lipid-related markers and arthritis. In this study, 18,683 participants in the NHANES 2001–2018 cohort were included. According to the study's results, the higher the seven lipid-related indicators, the higher the risk of arthritis, and the two were positively correlated. TyG, TyG-WC, TyG-WHtR, TyG-BMI, and VAI were all linearly associated with arthritis risk, while HOME-IR and LAP were non-linearly associated with arthritis risk. Finally, we performed ROC curve analysis and Logistic regression analysis, and the results showed that TyG-WC, TyG-WHtR, TyG-BMI, and LAP were all more accurate predictors of arthritis. The OR value of TyG-WHtR was the most advantageous, and it was the best predictor of arthritis among the seven indicators. All statistical models were adjusted for potential confounders, including age, sex, ethnicity, education, smoking status, hypertension, diabetes, coronary heart disease, stroke, and lipid profiles. Despite these adjustments, residual confounding from unmeasured variables (e.g., dietary patterns, physical activity) may persist. However, the robustness of our findings across quartile analyses and restricted cubic spline models supports the independent association between lipid-related indices and arthritis risk.

Although less research has been done on arthritis and IR using lipid-related indicators, the association of lipid-related indicators with arthritis and IR has been widely recognized. TyG, as an emerging and readily available indicator, has strong sensitivity and specificity in assessing insulin resistance (15), making it a reliable alternative biomarker for IR (16). At present, this indicator has been extended to clinical studies of diabetes, lung disease, cardiovascular disease, lung disease, and other major diseases (17–19). In previous studies of arthritis by scholars, TyG-related indicators have been recognized as an effective screening tool for IR in arthritis patients. Yan et al. (1) and Zhang et al. (20) have shown that the TyG index positively correlates with arthritis risk, and this study also obtained the same result. TyG and its derived indicators are positively correlated with arthritis risk. Our results also show that lipid-related indicators are more accurate in predicting IR than arthritis. In arthritis and IR, TyG-WC, TyG-WHtR, and TyG-BMI have higher sensitivity and specificity to disease than the TyG index alone. This is consistent with the findings of previous scholars (21, 22).

Some scholars have found that IR is closely related to the occurrence of arthritis (23, 24), and Mirjafari et al. (25) have shown that in inflammatory arthritis, there is a strong connection between insulin resistance and the presence of rheumatoid factor and anti-citrullinated protein antibodies. In addition, obesity promotes the production of adipokines in adipose tissue, which exacerbates autoimmunity and predisposes patients to metabolic syndrome, and low-grade systemic inflammation caused by metabolic syndrome can also induce arthritis, further emphasizing the critical role of IR in early arthritis (26). The TyG index takes into account two factors: glucose metabolism and lipid metabolism, while impaired glucose metabolism is a risk factor for arthritis (27), glucose metabolism can provide energy to immune cells to maintain autoimmune function, and insulin can regulate blood sugar indirectly affect the immune response of cartilage and synovium (28), or directly act on immune cells and affect their proliferation response and signal transduction to regulate the immune response (29). When IR occurs in the body, elevated blood sugar induces cellular stress, generating reactive oxygen species, which in turn induces inflammation (30). Hamada et al. showed that insulin inhibits the expression of ADAMTS4, matrix metalloproteinase 1 (MMP1), matrix metalloproteinase 13 (MMP13), and IL6 in fibroblast-like synoviocytes (FLS) in patients with osteoarthritis (6) These genes depend on tumor necrosis factor (TNF), which can damage cartilage, and insulin may protect cartilage by inhibiting TNF. If insulin resistance is present in the synovium, insulin resistance destroys this protective effect, leading to arthritis. The association of lipid abnormalities with arthritis has also been widely studied by scholars. One clinical study showed that patients who later developed arthritis had 4% and 17% higher serum cholesterol and triglycerides than the control group, respectively (31). Adipose tissue is a major source of inflammatory factors and is closely associated with arthritis (32). In the early stages of arthritis, when the synovium is not inflamed, the mitochondrial fatty acid β-oxidation in the synovium of arthritis is significantly impaired compared to the control group (33). In the later stages of arthritis, the inflammatory response of adipose tissue directly affects arthritis. Recent studies have shown that arthritis promotes a systemic inflammatory response, which affects adipose tissue, leading to IR and promoting lipolysis. Therefore, adipose tissue is an early target of this disease (34).

In this study, TyG-WHtR is the most accurate indicator for predicting arthritis. TyG-WHtR is composed of TyG indicators and WHtR indicators. TyG indicators were introduced above. WHtR indicators are waist circumference-to-height ratio, which is a new indicator for evaluating abdominal obesity. WHtR indicators may become a potential surrogate BMI indicator. Studies have shown that WHtR is most similar to the fat results measured by instruments in both men and women. WHtR can reflect the accumulation of visceral fat is less affected by skeletal muscle, and can more accurately estimate central obesity than BMI (35). Some studies have also shown that WHtR has a higher predictive ability for lipid abnormalities than TyG indicators.

## 5 Strength and limitation

This study has some merit. To our knowledge, we are the first to explore the impact of TyG and its derived and obesity-related indicators on arthritis risk. Our results show that TyG index, TyG-WC index, TyG-WHtR index, TyG-BMI index, HOMA-IR index, VAI index, and LAP index are all positively associated with arthritis. In particular, the TyG-WHtR index has the most efficient predictive power for arthritis. The sample size of this study is sufficient. We also adjusted for confounding factors to determine the independent association of 7 lipid-related indicators for arthritis.

This study has some limitations. First, the study's observational nature precludes conclusions about temporality or causality. We cannot determine whether elevated lipid indices precede arthritis onset or result from arthritis-related metabolic dysfunction. In addition, arthritis was defined broadly via self-report without distinguishing between osteoarthritis (OA) and rheumatoid arthritis (RA). These subtypes involve distinct pathophysiological mechanisms, potentially diluting or confounding subtype-specific associations. Although we adjusted for key covariates, residual confounding from factors like physical activity, diet, medication use (e.g., statins, DMARDs), or genetic predisposition cannot be excluded. In the future, we need to provide more robust data to support the impact of TyG and its derivatives and obesity-related indicators on arthritis.

## 6 Conclusion

In combination, the results we have obtained indicate a positive association between TyG-related parameters and obesity-related parameters with the risk of developing arthritis. Moreover, increased TyG-related and obesity-related parameters are significantly linked to a heightened susceptibility to arthritis. Among the 7 lipid-related parameters examined, the TyG-WHtR index emerges as the most precise and sensitive in forecasting arthritis, positioning it as a straightforward and readily computable clinical indicator for overseeing arthritis. To affirm the validity of our outcomes, meticulously planned longitudinal prospective studies in the future are imperative.

## Data Availability

The raw data supporting the conclusions of this article will be made available by the authors, without undue reservation.
